# Treatment of trigeminal ganglion neurons in vitro with NGF, GDNF or BDNF: effects on neuronal survival, neurochemical properties and TRPV1-mediated neuropeptide secretion

**DOI:** 10.1186/1471-2202-6-4

**Published:** 2005-01-24

**Authors:** Theodore J Price, Michael D Louria, Damaries Candelario-Soto, Gregory O Dussor, Nathanial A Jeske, Amol M Patwardhan, Anibal Diogenes, Amanda A Trott, Kenneth M Hargreaves, Christopher M Flores

**Affiliations:** 1 Department of Pharmacology, The University of Texas Health Science Center at San Antonio, USA; 2 Department of Endodontics, The University of Texas Health Science Center at San Antonio, USA; 3 Johnson and Johnson Pharmaceutical Research and Development, L.L.C., Welsh and McKean Roads, Spring House, PA 19477-0776, USA

## Abstract

**Background:**

Nerve growth factor (NGF), glial cell line-derived neurotrophic factor (GDNF) and brain-derived neurotrophic factor (BDNF) all play important roles in the development of the peripheral sensory nervous system. Additionally, these growth factors are proposed to modulate the properties of the sensory system in the adult under pathological conditions brought about by nerve injury or inflammation. We have examined the effects of NGF, GDNF and BDNF on adult rat trigeminal ganglion (TG) neurons in culture to gain a better understanding of how these growth factors alter the cytochemical and functional phenotype of these neurons, with special attention to properties associated with nociception.

**Results:**

Compared with no growth factor controls, GDNF, at 1 and 100 ng/ml, significantly increased by nearly 100% the number of neurons in culture at 5 days post-plating. A significant, positive, linear trend of increasing neuron number as a function of BDNF concentration was observed, also peaking at nearly 100%. NGF treatment was without effect. Chronic treatment with NGF and GDNF significantly and concentration-dependently increased 100 nM capsaicin (CAP)-evoked calcitonin gene-related peptide (CGRP) release, reaching approximately 300% at the highest concentration tested (100 ng/ml). Also, NGF and GDNF each augmented anandamide (AEA)- and arachidonyl-2-chloroethylamide (ACEA)-evoked CGRP release, while BDNF was without effect. Utilizing immunohistochemistry to account for the proportions of TRPV1- or CGRP-positive neurons under each growth factor treatment condition and then standardizing evoked CGRP release to these proportions, we observed that NGF was much more effective in enhancing CAP- and 50 mM K^+^-evoked CGRP release than was GDNF. Furthermore, NGF and GDNF each altered the concentration-response function for CAP- and AEA-evoked CGRP release, increasing the E_max _without altering the EC_50 _for either compound.

**Conclusions:**

Taken together, our results illustrate that NGF, GDNF and BDNF differentially alter TG sensory neuron survival, neurochemical properties and TRPV1-mediated neuropeptide release in culture. In particular, our findings suggest that GDNF and NGF differentially modulate TRPV1-mediated neuropeptide secretion sensitivity, with NGF having a much greater effect on a per neuron basis than GDNF. These findings are discussed in relation to possible therapeutic roles for growth factors or their modulators in pathological pain states, especially as these relate to the trigeminal system.

## Background

Among many trophic factors that act on sensory neurons, three have been studied extensively: nerve growth factor (NGF), glial cell line-derived neurotrophic factor (GDNF) and brain-derived neurotrophic factor (BDNF). During development, NGF, GDNF and BDNF, along with neurotrophin-3 (NT3), support the survival of subpopulations of sensory neurons through their cognate trk receptors [1-3]. In the adult, uninjured rat, receptors for NGF, GDNF and BDNF are found in partially distinct subpopulations of sensory neurons. NGF-responsive, trkA-containing neurons are mostly small diameter, contain the neuropeptide calcitonin gene-related peptide (CGRP), and are generally thought to have nociceptive properties [4,5]. The BDNF-responsive, trkB-containing population of sensory neurons is predominantly larger diameter and mechanosensitive [6], although there is considerable overlap with the trkA population [7]. GDNF receptors are more widespread, with as many as 60% of dorsal root ganglion (DRG) neurons containing c-ret, while GFR components are found throughout the c-ret population, as well as in c-ret negative neurons [8,9]. Additionally, sensory neurons that are postnatally dependent on GDNF for survival bind the isolectin B_4 _(IB_4_, [2]).

NGF is thought to play a primary role in the development and maintenance of several pro-algesic states. NGF produces sensitization of nociceptive responses in vivo and increases responsiveness to chemical stimuli associated with nociceptive neurotransmission in vitro [10-12]. NGF increases the expression of the pro-inflammatory neuropeptide CGRP [13] and increases substance P (SP) and CGRP content in sensory neurons [14-17]. NGF also promotes the development and maintenance of hyperalgesia following chronic constriction injury [18,19]. Additionally, NGF increases capsaicin (CAP) sensitivity both in vivo and in vitro [20-24]. In accordance with this increased CAP sensitivity, NGF increases the expression of the CAP- and noxious heat-sensitive ion channel vanilloid receptor type 1 (TRPV1) through the ras/p38 MAP kinase signaling pathway, thereby promoting thermal hyperalgesia [25,26]. Additionally, NGF has recently been shown to modulate TRPV1 by releasing the receptor from phosphotidylinositol-4,5-bisphosphate-mediated inhibition [27].

GDNF also plays a role in modulating nociceptive processing, but with contrasting in vitro and in vivo effects. In cultured DRG neurons, GDNF increases CAP sensitivity and TRPV1 expression [16,26] and increases neuropeptide content [16]. On the other hand, intrathecal injection of GDNF has no effect on C-fiber evoked outflow of SP and does not induce thermal hyperalgesia, while NGF does both [15]. This, coupled with the observation that GDNF-overexpressing mice do not develop thermal or mechanical hypersensitivity [28], suggests that GDNF might not lead to nociceptor sensitization in vivo. In fact, in nerve injured rats, GDNF has antihyperalgesic effects [29] and promotes the functional regeneration of DRG-spinal cord connections [30]. It is not understood how this dissociation of the in vitro and in vivo effects of GDNF is manifested.

Inflammation and nerve injury both increase BDNF content in DRG neurons and in the spinal cord, and this increase in BDNF is associated with the maintenance of a hyperalgesic state [31-36]. Furthermore, BDNF is released in the spinal cord upon noxious afferent stimulation [37,38], and peripheral CAP application increases BDNF release at central terminals of sensory neurons [39]. Hence, BDNF might act as a neurotransmitter in the pain pathway in adult animals [40]. The trophic properties of BDNF on adult sensory neurons, particularly nociceptors, are poorly understood.

In addition to CAP, a number of other TRPV1 agonists have been described. Among these are compounds that are also cannabinoid receptor agonists, including the endogenous cannabinoid anandamide (AEA, [41,42] and the synthetic AEA analogue arachidonyl-2-chloroethylamide (ACEA, [43]). AEA causes antinociception in vivo through central [44] and peripheral mechanisms [45,46]. It is not known, though, whether the endogenous production of AEA can reach sufficient concentrations under pathophysiological states to act as an endogenous activator of TRPV1. One potential mechanism whereby growth factors could contribute to the development of nociceptor sensitization is through the modulation of the efficacy or potency of AEA at TRPV1.Thus, growth factors might unmask conditions under which AEA could be capable of functioning as an endogenously produced TRPV1 agonist, potentially leading to neurogenic inflammation and thermal hyperalgesia.

The aim of the present work was to gain a more precise understanding of NGF-, GDNF- and BDNF-dependent alterations of cultured TG sensory neuron survival and phenotype, with attention to markers and functions that are related to nociception. Furthermore, by relating these findings to neurosecretion associated with pharmacological stimulation of TRPV1, we hope to present a rationale for differences in how these neurotrophins might contribute to altered nociception at the level of the TG sensory neuron. Moreover, there is a gap in knowledge concerning neurotrophic factor influence over TG neurons, as the vast majority of our understanding of how neurotrophins alter sensory neurons stems from studies of the DRG. The recent advances of CGRP receptor antagonists for the treatment of migraine [47,48] raises the possibility that manipulations which influence CGRP expression and/or secretion might be beneficial in the treatment of migraine or conditions related to the cerebral vasculature. A fuller understanding of the impact of NGF, GDNF and BDNF on TG neurochemical properties has the potential to lead to novel therapeutic strategies concerning disease states involving neuropeptide secretion in the trigeminal system, such as migraine.

## Results

### Effects of NGF, GDNF or BDNF treatment on neuronal survival in vitro

**Figure 1:**
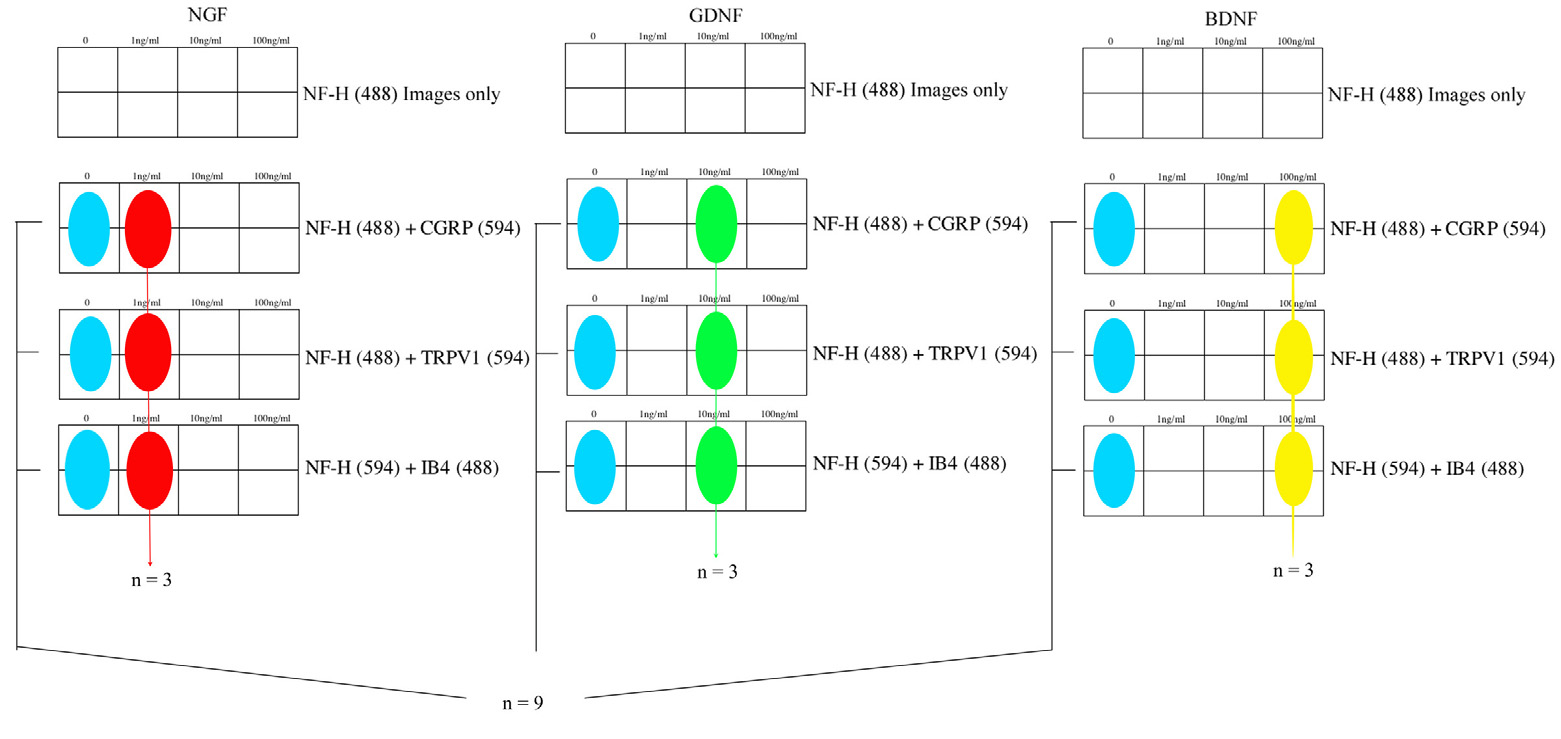
*Experimental design for neuronal counting*. 12 slides were utilized in these experiments, with 4 slides for each growth factor. The antibodies used on each slide are shown to the right of the slide along with the wavelength for the corresponding secondary antibody. For the 2 matching wells for each slide (growth factor concentration), the neuron numbers were averaged to give one observation (since the wells were not derived from independent cultures). Each slide contained a no growth factor control (blue), hence n = 9 for this condition. All other growth factors at 1 (i.e. NGF, red), 10 (i.e. GDNF, green) or 100 ng/ml (i.e. BDNF, yellow) are n = 3. The same slides were then utilized to ascertain the proportions of neurons expressing CGRP- or TRPV1-immunoreactivity or IB_4_-binding, as described in Methods. This figure refers to Figures 2 and 3 and Table 1.

**Figure 2:**
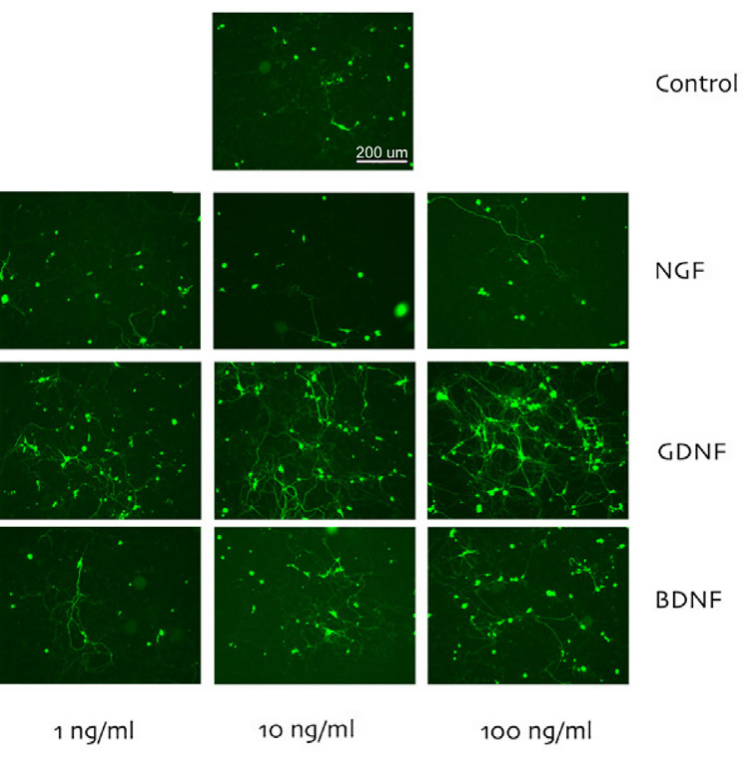
*Representative 20X photomicrographs of growth factor-treated TG neurons*. TG cultures were fixed and labeled for NF-H-immunoreactivity (green), and the 20X photomicrographs depicted here are representative of the 20 images taken per slide for each condition for neuron counting. The upper frame shows the no growth factor treated control with corresponding growth factor treatments shown for BDNF, GDNF and NGF at 1, 10 and 100 ng/ml concentrations.

**Figure 3:**
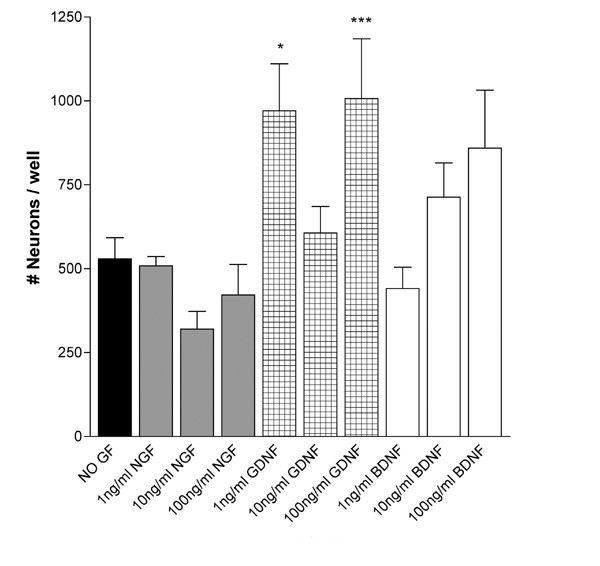
*NGF, GDNF and BDNF and TG neuronal surival*. All NF-H-immunoreactive neurons were counted for each growth factor-treated TG culture (n = 9 no growth factor; n = 3 all other conditions) and compared with the control (no growth factor-treated) TG cultures to assess neuronal survival at day 5 post-plating (* p < 0.05, *** p < 0.001).

TG neuronal cultures were plated at equal densities (~5000 neurons / well) on 8-well culture slides (experimental design shown in Figure 1). After five days of growth factor treatment, immunocytochemistry (ICC) for 200 kD neurofilament (NF-H) present in all sensory neurons [49] and not other ganglion cells (i.e., glia), was performed to determine the number of neurons in each well. Representative photomicrographs are shown in Figure 2. NGF did not significantly influence the number of neurons in culture following five days of treatment (Fig 3). On the other hand, GDNF significantly enhanced the number of neurons per well versus no growth factor treated cultures at both 1 and 100 ng/ml (Fig. 3). The enhanced neuronal survival seen with GDNF was biphasic, as 10 ng/ml GDNF did not significantly enhance neuronal survival. It should be noted that in GDNF-treated cultures we observed a robust sprouting of TG neurons, appearing far more robust than in NGF- or BDNF-treated cultures (although sprouting was still evident in these cultures).

### Effects of NGF, GDNF or BDNF treatment on TRPV1 mRNA and protein levels

**Figure 4:**
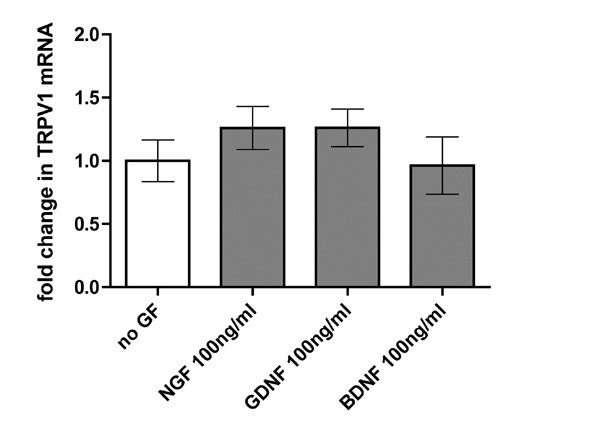
*Assessment of TRPV1 mRNA levels by Realtime PCR*. Levels of TRPV1 mRNA are depicted as fold change compared with no growth factor-treated cultures for each growth factor condition normalized to its corresponding GAPDH mRNA levels (n = 3).

**Figure 5:**
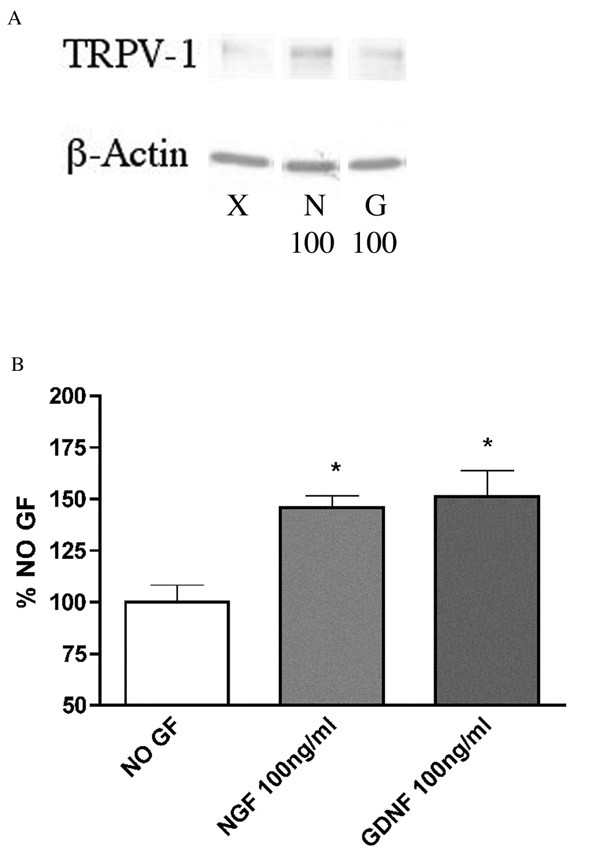
*Assessment of TRPV1 protein by Western Blot*. Panel A, 20 μg total protein was electrophoretically seperated per lane and transferred to membranes that were subsequently probed with an anti-TRPV1 antibody and then reprobed for standardization with β-actin (X = no growth factor; N 100 = NGF 100 ng/ml; G 100 = GDNF 100 ng/ml). Image is of a representative Western blot. Immunoreactivity to a protein corresponding to the size of the glycosyated form of TRPV1 was detected at ~130 kD. Panel B depicts alterations in TRPV1 protein levels standardized to β-actin (* p < 0.05, n = 3)

To directly assess changes in TRPV1 mRNA levels with growth factor treatment, quantitative, realtime PCR was conducted. NGF, GDNF or BDNF (100 ng/ml) did not significantly alter TRPV1 mRNA levels after 5 days of treatment (Fig. 4). To evaluate whether growth factor treatment might exert post-transcriptional regulation of TRPV1 gene expression, we next examined their effects on TRPV1 protein levels by Western blot. As shown in Fig. 5, a major band was detected with the TRPV1 antibody at ~130 kD, consistent with the glycosylated form of TRPV1 [25]. NGF and GDNF (100 ng/ml) each was demonstrated to have increased TRPV1 protein levels by approximately 50% compared with no growth factor-treated cultures (Fig 5). BDNF effects on TRPV1 protein levels were not assessed, because preliminary studies indicated that BDNF did not influence CAP-evoked CGRP release.

### Effects of NGF, GDNF or BDNF treatment on K^+^-evoked CGRP release and CGRP content

**Figure 6:**
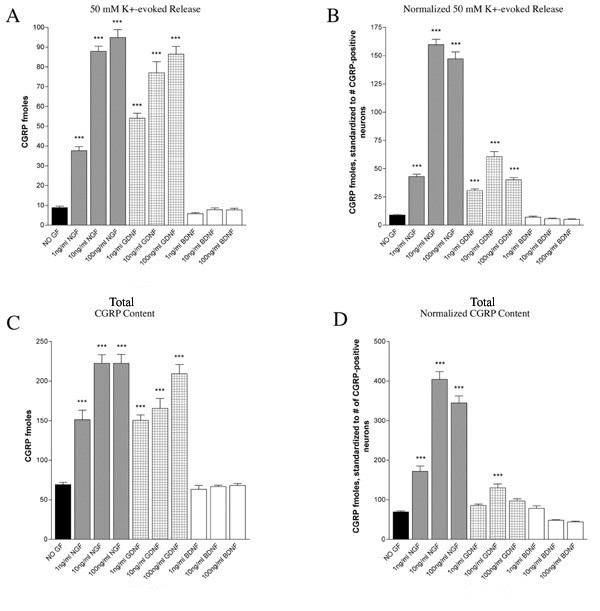
*50 mM K+ -evoked CGRP release and total CGRP content in TG cultures*. Panel A illustrates the effect of growth factor treatment on 50 mM K+ -evoked CGRP release, while panel B shows the data standardized to the number of CGRP-positive neurons per condition as a proportion of the no growth factor-treated cultures. Panel C shows total CGRP content by growth factor treatment, and again, panel D shows this data standardized to CGRP-positive neurons, as stated above (*** p < 0.001, n = 6).

We next examined the effect of growth factor treatment on K^+^-evoked CGRP release and total CGRP content in TG neuronal cultures. NGF treatment significantly and concentration-dependently increased 50 mM K^+^-evoked CGRP release at every concentration, peaking (10.5-fold increase vs. control) at 100 ng/ml (Fig. 6A). Similarly, GDNF significantly and concentration-dependently increased K^+^- evoked release at every concentration, again peaking (9.7-fold increase vs. control) at 100 ng/ml. On the other hand, BDNF did not alter K -evoked CGRP release.

**Figure 7:**
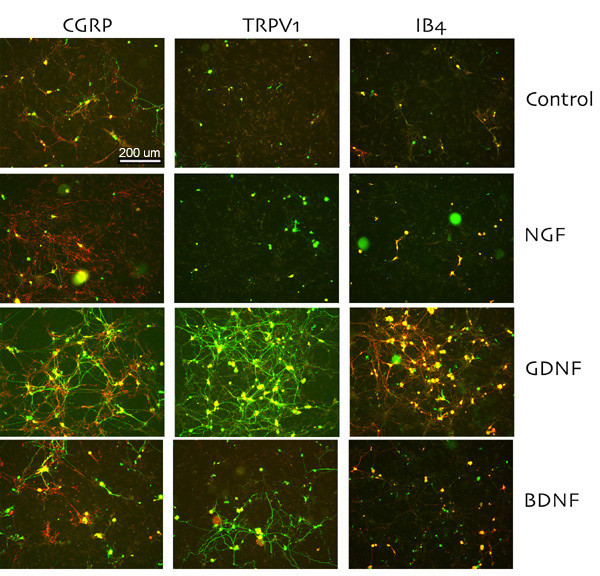
*Representative 20X photomicropgraphs of colocalization of sensory neurons markers with NF-H in growth factor-treated TG neurons*. Immunoreactivity for CGRP and TRPV1 and staining for IB_4_-binding sites (red) was assessed following immunocytochemistry for NF-H (green) to assess the proportion of neurons expressing these population markers. Representative 20X photomicrographs of each growth factor at 100 ng/ml for CGRP (left), TRPV1 (middle) and IB_4 _(right) are shown as well as control (no growth factor-treated) TG cultures (top panels). All cell bodies containing both NF-H-immunoreactivity and CGRP- or TRPV1-immunoreactivity or IB_4_-binding appear yellow from the overlay of the red with green. In no cases were neurons observed that contained CGRP- or TRPV1-immunoreactivity or IB_4_-binding without the co-presence of NF-H-immunoreactivity.

Because there were differences in neuron numbers between growth factor conditions, we examined under each condition the proportion of neurons that were positive for CGRP, TRPV1 or IB_4_. Representative photomicrographs for each of these conditions are shown in Figure 7. The proportion of neurons positive for CGRP, TRPV1 or IB_4 _are shown in Table 1. In no cases were CGRP- or TRPV1-immunoreactive or IB_4_-binding neurons not likewise immunoreactive for NF-H. We then utilized the proportion of CGRP-expressing neurons under NGF, GDNF or BDNF treatment conditions to normalize K^+^- evoked CGRP release to the number of CGRP-immunoreactive neurons under each condition, respectively (see *Methods *for normalization calculation). Following this transformation, NGF treatment still augmented K^+^-evoked CGRP release at every concentration tested, with the magnitude of the effect reaching 18 times that of control (Fig 6B). While GDNF treatment again enhanced K^+^-evoked CGRP release at all concentrations following normalization, its peak effect was relatively lower. BDNF treatment continued not to have an effect following normalization to the number of CGRP-immunoreactive neurons.

Much like K^+^-evoked CGRP release, total CGRP content was increased significantly by NGF and GDNF treatment, while BDNF had no effect (Fig. 6C). Both NGF and GDNF increased CGRP content concentration-dependently at every concentration (p < 0.001), with peaks at 10 ng/ml (3.2-fold increase) for NGF and 100 ng/ml (3.0-fold increase) for GDNF (Fig. 6C). Again, we normalized these data to CGRP-immunoreactive neurons to assess changes in relation to alterations in CGRP-immunoreactive neurons between the growth factor conditions. Interestingly, the GDNF augmentation of CGRP release appeared to be due primarily to the large increases in neuron survival, because only the 10 ng/ml GDNF condition was significantly greater than control (p < 0.001), but still substantial lower compared with the untransformed lysis figures (Fig. 6D). On the other hand, normalization led to a further augmention in the effect of NGF on total CGRP content, up to a 5.8-fold increase. BDNF treatment did not alter the normalized CGRP content.

### Effects of NGF, GDNF or BDNF treatment on CAP-evoked CGRP release

**Figure 8:**
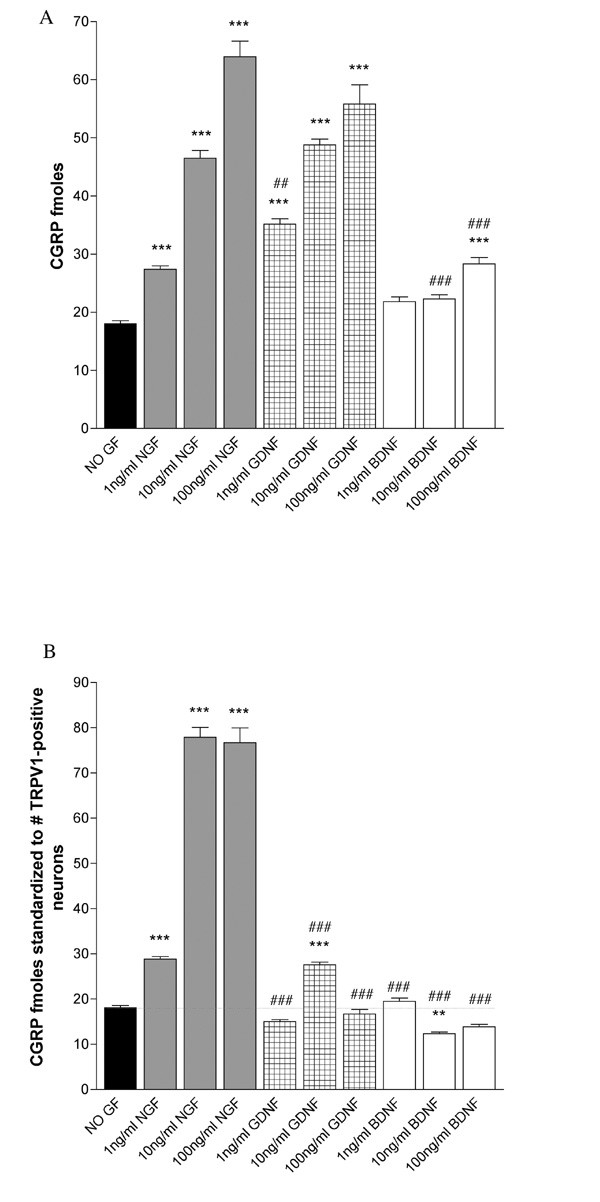
*Growth factor treatment increases CAP (100 nM)-evoked CGRP release in TG cultures*. Panel A illustrates the total amount of CGRP released by 10 min treatment with capsaicin for each growth factor. Panel B illustrates the same data standardized to the number of TRPV1-positive neurons under each growth factor condition (** p < 0.01, *** p < 0.01 for comparisons to no growth factor treated controls; ## p < 0.01, ### p < 0.001 for comparisons to NGF at the equivalent growth factor concentration by two-way ANOVA with Bonferroni post-test; n = 6).

To assess the effect of growth factor supplementation on the neurosecretory function of nociceptors in culture, we evaluated CAP-evoked CGRP release. Treatment with 100 nM CAP for 10 min led to a significant enhancement of CGRP release over baseline under all conditions. Treatment of cultures with NGF or GDNF resulted in a concentration-dependent enhancement of this effect, with significant increases in CAP-evoked CGRP release at 1, 10 and 100 ng/ml. BDNF also enhanced the ability of CAP to evoke CGRP release; however, the effect was only seen at the 100 ng/ml concentration. In every case, GDNF and NGF (except 1 ng/ml NGF) supplementation significantly enhanced CAP-evoked CGRP release over the same concentration of BDNF (Fig 8A).

To determine to what extent the enhancement of CAP-evoked CGRP release in response to growth factor treatment might derive from increased neuronal survival and/or up-regulation in the proportion of neurons expressing TRPV1, release data were normalized to the number of TRPV1-immunoreactive neurons (Fig. 8B; TRPV1 neuron proportions shown in Table 1). In this case, NGF treatment still significantly increased CAP-evoked CGRP release over no growth factor treatment at 1, 10 and 100 ng/ml. However, this normalization procedure led to a relative reduction in the GDNF effect on CAP-evoked CGRP release, such that only the 10 ng/ml condition reached a significant increase in CGRP outflow. After normalization, BDNF treatment had no significant effect on CAP-evoked CGRP release at 1 or 100 ng/ml and actually reduced the amount of release at 10 ng/ml. Moreover, following normalization, NGF-treated neurons displayed a significantly higher CGRP release than either GDNF- or BDNF-treated neurons at every concentration of growth factor tested (Fig 8B).

**Figure 9:**
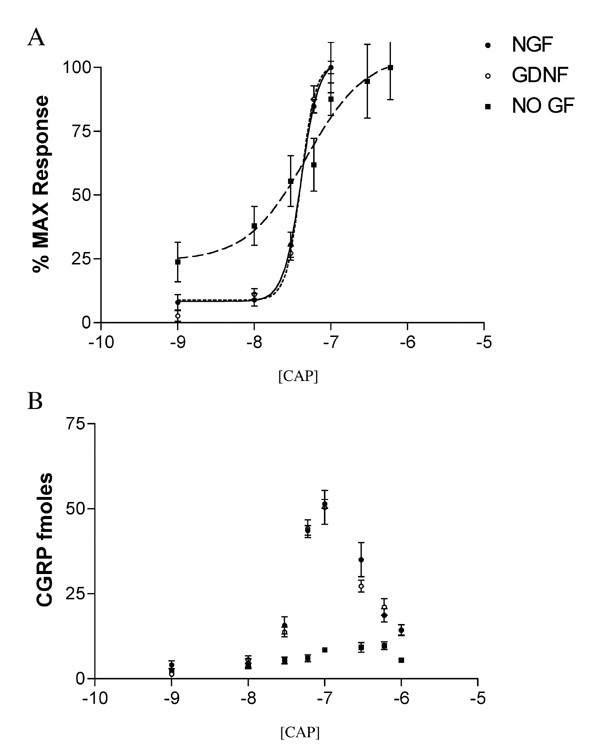
*NGF and GDNF alter capsaicin-evoked CGRP release*. Concentration-response functions (molar, log units) are shown for capsaicin-evoked CGRP release in TG cultures maintained in 100 ng/ml NGF, 100 ng/ml GNDF or no growth factor conditions (n = 6). Panel A illustrates the maximum relative effect of capsaicin under each condition matched to the maximum evoked-CGRP release for that given condition. Panel B depicts the CAP-evoked CGRP release data in terms of CGRP released in fmoles in the presence or absence of NGF or GDNF to illustrate the magnitude of increases in E_max _and the biphasic nature of the capsaicin-evoked CGRP concentration-response function.

Because treatment with either GDNF or NGF induced a large increase in 100 nM CAP-evoked CGRP release, we assessed the effect of supplementation with these growth factors on the receptor pharmacodynamics of this response compared with no growth factor supplementation. GDNF or NGF treatment significantly increased the Hill slope of the concentration-response function from 1.12 ± 0.47 in the no growth factor condition to 3.760 ± 0.057 for 100 ng/ml NGF and to 4.48 ± 0.90 for 100 ng/ml GDNF (Fig 9A) but had no effect on the EC_50 _(41 nM for GDNF or NGF and 47 nM for no growth factor). GDNF (100 ng/ml) or NGF (100 ng/ml) treatment caused a significant, five-fold increase in the E_max _of the CAP concentration-response curve (Fig. 9B). A common feature of CAP concentration-response functions is that they frequently exhibit an inverted U-shape, likely attributable to TRPV1 desensitization at higher concentrations. Thus, whereas the concentration-response function in the absence of growth factor displayed desensitization only at 1 uM, that for either GDNF- and NGF-treated TG cultures showed desensitization of the CAP-evoked CGRP response at concentrations above 100 nM (Fig. 9B).

### Effects of NGF, GDNF or BDNF treatment on AEA- and ACEA-evoked CGRP release

**Figure 10:**
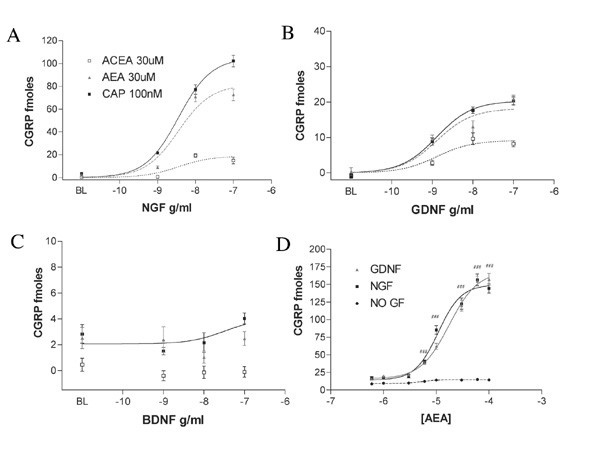
*The effect of growth factor treatment on AEA- and ACEA-evoked CGRP release*. TG neurons were grown in the presence of the indicated concentrations of NGF (panel A), GDNF (panel B) or BDNF (panel C) for five days and then exposed to 100 nM CAP, 30 μM AEA or 30 μM ACEA for 10 min, and the released CGRP was quantified (n = 12). Panel D, TG neurons were grown in the absence of growth factors or in the presence of 100 ng/ml NGF or GDNF for 5 days and exposed to the indicated concentrations of AEA (molar, log units) to assess CGRP release (### p < 0.001; GDNF and NGF vs. no growth factor; two-way ANOVA, Bonferroni post-test, n = 6).

To test whether growth factor-mediated enhancement of neuropeptide release might be generalizable to other TRPV1 secretagogues, we evaluated the dual cannabinoid-vanilloid agonists AEA and ACEA. As in the case with CAP, the effects of AEA and ACEA on evoked CGRP release were augmented by supplementation of TG cultures with NGF (Fig. 10A) or GDNF (Fig. 10B). While TG neurons not treated with growth factors were largely unresponsive to AEA or ACEA, in terms of CGRP release, treatment of cultures with either NGF or GDNF for 5 days at 1, 10 or 100 ng/ml caused a concentration-dependent increase in CGRP release evoked by 30 μM AEA or ACEA. Furthermore, the estimated EC_50 _values for each of the individual growth factors to increase evoked CGRP release were equivalent for CAP, AEA and ACEA (NGF = 3.5 ng/ml; GDNF = 1.3 ng/ml). In contrast, BDNF supplementation did not augment AEA- or ACEA-evoked CGRP release, while a small, yet significant, increase in CAP-evoked CGRP release was observed with increasing BDNF concentration (Fig. 10C). When TG neurons were supplemented with either 100 ng/ml NGF or GDNF, the E_max _and EC_50 _values for AEA did not change (F = 2.082_(4,85)_) ; however, when compared with non-growth factor-treated TG neurons, the E_max _was significantly augmented (Fig. 10D).

## Discussion

This study demonstrates that NGF, GDNF and BDNF differentially influence neuronal survival, neuropeptide content and stimulated secretion of TG neurons in culture. GDNF significantly augments TG neuronal survival, while both NGF and GDNF modulate CAP sensitivity, and alter the pharmacodynamics of the concentration-response function for CAP-evoked CGRP release. Furthermore, NGF and GDNF increased the releasable pool and total content of CGRP while increasing TRPV1 protein, without increasing its mRNA. These data support the hypothesis that GDNF and NGF, but not BDNF, alter CAP sensitivity in cultured TG neurons, and taken together, suggest that the differential effects of NGF and GDNF in vitro may reflect their differential effects in vivo, particularly with regard to TRPV1-mediated nociception.

GDNF, but not NGF or BDNF significantly increased the number of neurons present in TG culture 5 days post-plating. The finding that this effect of GDNF was biphasic, as the 10 ng/ml concentration did not enhance the number of neurons in TG culture, suggests the possible involvement of different receptors with different concentrations of GDNF. Multiple receptors exist in the GDNF receptor family, and they function in concert with the protein c-ret. GDNF binds most readily to GFR alpha-1, but also binds to GFR alpha-2 / c-ret heterodimers [50]. GFR alpha-1 and -2 are expressed in sensory ganglia and are found mostly in IB_4_-binding neurons [51] that also contain c-ret [8]. Notably, it has been suggested that GFR alpha-1 and GFR alpha-2-containing neurons in the adult rat make up two distinct populations in the DRG within the IB_4_-binding class [8]; hence, the effects observed here may be due to GDNF acting through its high affinity GFR alpha-1 site at the 1 ng/ml concentration and through either or both GFR alpha-1 and GFR alpha-2 / c-ret, for which it has a lower affinity, at the 100 ng/ml concentration. While the observation here that GDNF promotes survival is a novel finding for rat TG sensory neurons in primary culture, the neuroprotective effects of GDNF are well documented. GDNF is protective against the loss of dopaminergic neurons in animal models of Parkinson's disease [52,53], and GDNF reduces the number of apoptotic bodies in DRG explants from adult mice [54]. GDNF also rescues the reduction of P2X3 expression that occurs in the DRG following axotomy [55].

Although BDNF and NGF did not significantly increase the number of TG neurons in culture, we did observe a significant, linear trend for increased neuron survival as a function of increasing BDNF concentrations, an observation not present in NGF-treated TG cultures. Hence, BDNF and GDNF both promoted the survival of TG neurons in vitro. Primary cultures generated here were grown in the presence of mitotic inhibitors, which greatly reduce the supportive glial cells and the trophic factors they normally provide TG neurons in the native ganglia (although some of these cells are still present). Removal of astroglial support promotes apoptosis in cerebral neuronal cultures, an effect which is reversed by addition of GDNF or BDNF, but not NGF, to the culture medium [56]. The addition of GDNF or BDNF to the culture medium here may have re-supplied, at least partially, the withdrawn glial-supplied trophic factors that maintain sensory neurons in vivo, thereby increasing the number of neurons in culture at 5 days. It should be noted, however, that despite the significant effects of the growth factors observed in this study on neuronal survival, from an original density of nearly 5,000 neurons / well in the original culture homogenate, only about 10% of neurons could be counted at 5 days post-platting, in the control condition. A maximum of just over 20% were counted in the 100 ng/ml GDNF treated TG cultures. Therefore, it stands to reason that GDNF and BDNF are supporting the survival of certain classes of sensory neurons that are not supported either without growth factors or with NGF alone.

The proportions of TG neurons in culture that expressed CGRP- or TRPV1-immunoreactivity or IB_4_-binding sites were assessed with attention to how these proportions changed in the presence of NGF, GDNF and BDNF. Notably, the proportion of sensory neurons in vitro that expressed CGRP (~65%) was much larger than the known proportion of CGRP-containing neurons in native TG (~35%, [57]). This likely indicates that the culturing process conditions are either selectively preserving peptidergic neurons or that normally non-peptidergic neurons novelly express CGRP in culture. On the other hand, discrepancies on reports of the percentage of IB_4_-binding neurons in native TG, ranging from ~35–60% [58,59] make it difficult to assess whether there is an increased proportion of IB_4_-binding neurons in culture; however, our data indicate that this proportion is at least in the upper range, if not greater than native TG. Furthermore, a probable significant overlap exists between CGRP-immunoreactive and IB_4_-binding neuronal populations observed here, as both were found in the majority of TG neurons in culture, in agreement with the demonstration that these populations overlap significantly in native, adult rat TG and DRG (Price and Flores, unpublished observations).

We observed that a greater proportion of TRPV1-immunoreactive neurons were present with GDNF supplementation (nearly 30% increase), again indicating either that GDNF preferentially supports the survival of TRPV1-expressing sensory neurons or that neurons that do not normally express TRPV1 begin expressing TRPV1 when GDNF is included in the culture medium. The latter proposition is supported by the finding that peripheral treatment with anti-GDNF antibodies suppresses the novel expression of TRPV1 in IB_4_-binding neurons following peripheral inflammation [60]. The percentage of TRPV1-expressing neurons in TG cultures not treated with growth factors was essentially equivalent to the percentage in native TG [57]. This finding indicates that, not only does GDNF promote survival of TG neurons in culture, but also enriches for TRPV1-expressing neurons, suggesting that GDNF might be preferentially neuroprotective for sensory neurons in adult animals that express TRPV1.

We have also illustrated that chronic application of NGF or GDNF, but not BDNF (except at high concentrations, and to a much lesser degree), increases CAP-evoked CGRP release from TG neurons in vitro. NGF and GDNF each increased TRPV1 protein, and both upregulated the CGRP content of TG neurons. Both the NGF- and GDNF-induced upregulation of TRPV1 appears to be translationally regulated, as neither of these growth factors altered TRPV1 mRNA levels. NGF, in the setting of inflammation, is known to increase TRPV1 protein, but not mRNA, through activation of the p38/MAP kinase pathway [25,26], and NGF-mediated upregulation of TRPV1 is blocked by over-expression of dominant-negative ras [26]. Interestingly, the study by Bron et al. (2003) indicated that GDNF is also able to upregulate TRPV1 expression; although this conclusion was based on immunofluorescence and cobalt uptake assays, it is consistent with our direct demonstration of GDNF-induced upregulation of TRPV1 protein by Western blot. After normalization to the number of neurons that express TRPV1 and CGRP, we found that NGF had a much greater effect on CAP-evoked CGRP release and neuropeptide content, on a per cell basis, than did GDNF. On the other hand, GDNF increased the proportion of neurons in culture that express TRPV1, such that GDNF-maintained cultures contained a higher number of TRPV1-expressing neurons compared with NGF-maintained cultures. Hence, our findings suggest that NGF increases CAP responsiveness in individual cultured TG neurons. On the other hand, GDNF appears to increase the responsiveness of the in vitro population by altering the proportion of TRPV1 neurons and upregulating the releasable pool of CGRP, as evidenced by the persistent increase in 50 mM K^+^-evoked CGRP release after normalization. This difference in the ability of GDNF and NGF to enhance individual neuronal neuropeptide content and CAP responsiveness could partially explain why NGF induces hyperalgesia [10,12,19,61] while GDNF does not [15,28,29]. Our findings suggest that while GDNF might play a role in maintaining CAP sensitivity in vitro, its effects in vivo might be of a preservative nature that prevents the development of pain exacerbation following experimental manipulation. Furthermore, NGF and GDNF might differentially/predominantly subserve two of the main physiologic processes following injury: hyperalgesia to protect the organism from further injury (NGF) and regeneration/repair to restore function (GDNF).

While it has been shown, using a variety of dependent measures, that both NGF and GDNF increase CAP responsiveness [16,17], this is the first demonstration that these growth factors qualitatively alter the pharmacodynamics of the neuronal response to CAP in TG neurons. The increase in the Hill slope of the CAP response following exposure to NGF or GDNF indicates that these growth factors induce positive cooperativity, possible at the level of TRPV1. While the mechanism underlying this effect is not known, it could involve an alteration in post-translational modifications and/or protein interactions of TRPV1 in response to NGF or GDNF. NGF or GDNF also decreased the concentration of CAP necessary to induce tachyphylaxsis compared with control cultures. These findings indicate that TRPV1-mediated sensory neuron desensitization might be a more efficacious therapeutic strategy in pathologies known to be associated with increased levels of NGF and/or GDNF. While we are unaware of any data linking GDNF with migraine, increased cerebrospinal fluid levels of NGF are associated with chronic headache [62]. Interestingly, a TRPV1 targeted approach has been utilized in a clinical trial for migraine treatment in which intranasal civamide (a TRPV1 agonist) was shown to be effective for acute treatment of migraine [63], indicating that TRPV1 agonists might be employed in this condition, possibly to desensitize CGRP-containing TG nerve endings.

Similarly to CAP-evoked CGRP release, the ability of AEA and ACEA to evoke release was concentration-dependently augmented by NGF or GDNF supplementation but was unaltered by BDNF. Moreover, the respective potencies of NGF and GDNF to enhance the CGRP release in response to CAP, AEA and ACEA were equivalent. This suggests that the pharmacology of CAP, AEA and ACEA at TRPV1, at least with respect to neuropeptide secretion, is similarly regulated in the presence of either of these growth factors. Insofar as NGF and GDNF have been implicated in the development of nociceptor sensitization in a number of pathological states, it should be considered that endocanninoids are apt to have enhanced TRPV1-mediated peripheral neuromodulatory effects under these conditions.

## Conclusions

Taken together, our results illustrate that NGF, GDNF and BDNF differentially alter sensory neuron survival, neurochemical properties and TRPV1-mediated neuropeptide release of TG neurons in culture. GDNF and, to a lesser extent, BDNF promote survival of TG neurons and GDNF enhances the proportion of neurons that exhibit TRPV1-immunoreactivity. GDNF or NGF enhanced CAP-, AEA- and ACEA-evoked CGRP release from TG neurons in vitro and increased TRPV1 protein, likely through translational regulation, and overall CGRP content. On the other hand, our findings suggest that GDNF and NGF differentially modulate TRPV1-mediated neuropeptide secretion sensitivity, with NGF having a much greater effect on a per neuron basis, providing a possible explanation for why NGF promotes thermal hypersensitivity in vivo while GDNF apparently does not. Although the present studies were conducted on cultured neurons under artificially controlled conditions, these findings contribute to a growing body of work concerning neurotrophin modulation of the properties of nociceptors. Thus the results described here have implications for advancing our understanding of how this system might be advantageously manipulated in a therapeutic setting especially as it concerns the TG system.

## Methods

### Experimental chemicals

Capsaicin (CAP, 8-methyl-N-vanillyl-trans-6-nonenamide) was from Fluka-Aldrich (St Louis, MO). AEA (*N*-(2-hydroxyethyl)-5Z,8Z,11Z,14Z-eicosatetraenamide, in water soluble emulsion), and ACEA (*N*-(2-chloroethyl)-5Z,11Z,14Z)-eicosatetraenamide were from Tocris (Ellisville, MO). Recombinant rat GDNF and recombinant human (100% homologous to rat) BDNF were from Sigma (St Louis, MO). Rat NGF was from Harlan (Indianapolis, IN). All other chemicals were from Sigma, unless otherwise stated.

### TG culture

Adult, male Sprague-Dawley rats weighing 250–300 g were used in this study. All procedures utilizing animals were approved by the Institutional Animal Care and Use Committee of The University of Texas Health Science Center at San Antonio and were conducted in accordance with policies for the ethical treatment of animals established by the National Institutes of Health. Animals were euthanized by decapitation and their TGs were rapidly dissected (~30 s) and placed in ice-cold Ca^++^- and Mg^++^- free Hank's balanced salt solution (HBSS, Gibco, Carlsbad, CA). TGs were enzymaticaly digested for 30 min with 5 mg/ml collagenase followed by 25 min with 0.1% trypsin type IX supplemented for the last 10 min with 10 units of DNase I (Roche, Indianapolis, IN). TG cell suspensions were then centrifuged at 2000 RPM for 2 min, vortexed briefly and centrifuged again. They were then resuspended in basal culture medium containing high glucose Dulbeco's Modified Eagle's Medium (DMEM, Gibco), 1X pen-strep (Gibco), 1X glutamine (Gibco) and 3 μg/mL 5-FDU and 7 μg/mL uridine as mitotic inhibitors. TG cell suspensions were gently triturated with a Pasteur pipette followed by successive triturations through 19- and 23-gauge needles. TG cell suspensions were then transferred to a separate container and adjusted to the total volume needed for plating at a density of ~5000 neurons / well. The appropriate growth factors were added to this suspension prior to plating.

### Immunocytochemistry (ICC) and Image Aquisition

TG cultures were first washed in PBS and fixed for 1 hr in 3.7% formaldehyde in PBS. Next, TG cultures were washed 3 times in PBS and permeabilized in PBS containing 10% normal goat serum (NGS, Gibco) and 0.2% Triton X-100 (Sigma) for 1 hr. Finally, TG cultures were blocked 3 × 10 min in PBS containing 10% NGS and then exposed to NF-H mouse-monoclonal antibody (1:300; Sigma) overnight at 4°C. Primary antisera were then washed off and goat anti-mouse Alexa-Fluor-488 (1:300, Molecular Probes, Eugene, OR), or goat anti-mouse Alexa-Fluor-594 (1:300) was applied for 1 hr at room temperature. For double-labeling either CGRP rabbit polyclonal (1:750, Peninsula Labs) or TRPV1 guinea pig polyclonal (1:3000, Neuromics) or IB_4_-conjugated to Alexa-Fluor-488 (1:1000, Molecular Probes) was then added overnight at 4°C. CGRP or TRPV1 antisera were followed by goat anti-rabbit Alexa-Fluor-594 (1:300) for 1 hr at room temperature. All images were acquired using a Nikon E600 microscope (Melville, NY) equipped with a Photometrics SenSys digital CCD camera (Roper Scientific, Tucson, AZ) connected to a computer equipped with Metamorph V4.1 image analysis software (Universal Image Corporation, Downingtown, PA). Twenty 20X images were taken of each well to capture the complete cellular area for neuron counts. For these experiments, all neurons displaying fluorescent signal above background were counted as positive for the specific marker. This was defined by scaling the image, using Metamorph's built-in scaling feature, to an average pixel value for negative neurons in the case of TRPV1, CGRP and IB_4 _(no scaling was performed for NF-H ICC) and establishing all neurons as positive that were above that threshold. For double labeling experiments, the thresholded images were then overlaid and analyzed for the presence of both signals to assess colocalization.

### Neuronal Counting (Survival)

TG cell suspensions (~5000 neurons/well) were added to 8-well poly-D-lysine Lab-Tek II chamber slides (Nalge/Nunc, Naperville, IL). Neuron density for plating was determined by counting neurons with a hemacytometer with Neubauer rulings and the cultures for each slide were generated independently. Neurons were easily distinguished from other cell types for plating density measurements due to their large size and opaqueness. Medium was changed after 24 hr and 72 hr, and on day 5, ICC was performed (as described above). In total, 12 chamber slides were utilized. The experimental design is shown in figure 1 with 4 slides each for NGF, GDNF and BDNF. Neurons on the NF-H alone slides were not counted as these slides were used only for images shown in figure 2. To assess neuronal survival, all NF-H- immunoreactive neurons were counted for each growth factor concentration and are presented as mean ± SEM. The 2 matching wells for each slide were averaged and this average was used for 1 observation (as described in Fig 1). For colocalization studies, the previously generated NF-H-immunoreactive neuron counts for each well were followed by counting of CGRP- or TRPV1-immunoreactive or IB_4_-binding neurons in the same wells to generate the proportion of neurons expressing these markers. The total for the 2 matching wells were summed to yield the final proportion. At least 500 NF-H-immunoreactive neurons were counted under every growth factor condition to calculate proportions of neurons expressing the three markers examined. In no cases were CGRP-, TRPV1- or IB_4_-binding neurons not likewise positive for NF-H. Colocalization is presented only as an overall percentage.

### Realtime PCR

TG cultures were prepared on 48-well poly-D-lysine pre-coated plates (Becton Dickinson, Franklin Lakes, NJ) at an initial density of ~5000 neurons/well. For each 48 well plate 12 wells received no growth factor, 12 received 100 ng/ml NGF, 12 received 100 ng/ml GDNF and the remaining 12 received 100 ng/ml BDNF. A total of 3 plates were utilized and each culture plate was generated independently. Following 5 days of culture, RNA was extracted from TG cultures using a ToTALLY RNA (Ambion, Austin, TX) total RNA extraction kit and each of the 12 wells per condition were pooled together to yield sufficient RNA. RNA samples were subsequently treated with DNA-free DNAse (Ambion) for removal of trace amounts of DNA. RNA concentrations were determined by UV absorbance, and RNA samples were then diluted to equal concentrations in TE buffer. For realtime PCR assessment of TRPV1 mRNA levels, samples were loaded in triplicate in 96-well reaction plates with each sample containing 150 ng RNA, 2X RT-PCR Taqman Master Mix, 40X Multiscribe and RNase Inhibitor solution, forward primer (tcc agt caa gcc cca cat c), reverse primer (tcc gag tca ccc ttc cca) and Taqman probe (6FAM tca cta cca gga gtc gta ccc ggc ttt TAMRA) (all 300 nM, all reagents Applied Biosystems, Foster City, CA) in a final reaction volume of 50 μL. Controls were run concomitantly using the same reaction recipe with a rodent GAPDH control kit (Applied Biosystems), primers and probe at 50 nM. Reactions were run on an ABI Prism 7700 (Applied Biosystems) with an initial RT step of 48°C for 30 min followed by a 95°C 10 min denaturation step and then a repeating denaturation, extension cycle of 95°C for 15 s and 60°C for 1 min for 55 cycles in order to reach a full plateau for all samples. All data were normalized to GAPDH mRNA levels to account for any variation in RNA concentrations between samples.

### Western blotting

TG cultures were prepared on 48-well poly-D-lysine pre-coated plates at an initial density of ~5000 neurons/well. For each 48 well plate 12 wells received no growth factor, 12 received 100 ng/ml NGF, 12 received 100 ng/ml GDNF and the remaining 12 received 100 ng/ml BDNF. A total of 3 plates were utilized and each culture was generated independently. After five days, total protein was extracted by first lysing cells with lysis buffer (1 mM Na pyrophosphate, 50 mM HEPES, 1% Triton X-100, 50 mM NaCl, 50 mM NaF, 5 mM EDTA and 1 mM Na orthovanadate) supplemented with 1% protease inhibitor cocktail and then homogenizing the combined lysate from the 12 wells per condition by pumping it through a 25 gauge needle 20 times. Extracted proteins were cleared of nuclei and cellular debris by spinning the homogenate at 1000 × g for 5 min at 4°C. Protein levels were then measured by the Bradford method. Proteins were run at a concentration of 20 μg/lane on a 12.5% SDS-PAGE gel and transferred to Immobilon – P membranes (Millipore). Membranes were blocked in 5% dry milk for 1 hr and then exposed to rabbit anti-TRPV1 antibody (Neuromics, Minneapolis MN), at a concentration of 1:1000, overnight at 4°C. Membranes were then incubated with donkey anti-rabbit horseradish peroxidase-linked secondary antibody (Amersham, 1:5000) for 1 hr followed by ECL Western blotting detection for 1 min (Amersham). Blots were next exposed to film and subsequently scanned on a flatbed scanner. To control for protein loading, membranes were then stripped and reblotted for β-actin. Images were assessed for changes in TRPV1 protein using NIH image and normalized to β-actin protein levels.

### CGRP release and CGRP content

All experiments were performed in 48-well poly-D-lysine pre-coated plates. Data shown are representative of at least 3 independently conducted CGRP release experiments with consistent results and neurons were plated at the same density as indicated for survival, ICC, realtime PCR and Western blotting experiments (~5000 neurons/well). Culture medium was changed at 24 and 72 hr, and all CGRP assays were performed on day 5. TG cultures were washed free of culture medium by 2 successive washes with release buffer (Hank's balanced salt solution (Gibco) supplemented with 10.9 mM HEPES, 4.2 mM sodium bicarbonate, 10 mM dextrose and 0.1% bovine serum albumin (BSA), pH 7.4). Growth factors were not included in the release buffer. Following washing, TG cultures were exposed for 10 min to the indicated concentrations of CAP, AEA or ACEA or to 50 mM K^+ ^buffer (containing 2.5 mM CaCl_2_, 50 mM KCl, 1.2 mM MgCl_2_, 90 mM NaCl, 25 mM NaHCO_3_, 1 mM NaH_2_PO_4_, 10 mM dextrose, 15 mM Hepes, 16 uM thiophan and 0.1% BSA at pH = 7.4), after which the CGRP-containing supernatant was removed and transferred to glass culture tubes (Fisher). Content was assessed by hypotonic lysis with deionized H_2_O supplemented with 1% protease inhibitor cocktail (Sigma) for 30 min. CGRP release or content for each well was subsequently measured by radioimmunoassay.

### CGRP radioimmunoassay

Following culture release assays, individual aliquots of the superfusate (0.5 ml) were incubated with a C-terminally directed anti-CGRP antiserum (kindly donated by Dr Michael Iadarola, NIDCR, NIH, Bethesda, MD, USA). After 24 h, 100 μL of [125^I^]-CGRP_28–37 _(approximately 20000–25000 cpm) and 50 μL of goat anti-rabbit antibody conjugated to ferric beads were added. Following another 24 h, bound peptide was separated from free peptide via immunomagnetic separation (PerSeptive Biosystems, Framingham, MA, USA). All incubations were carried out at 4°C. The minimum detection limit for this assay is approximately 1–2 fmol per tube, with 50% displacement occurring at 20–40 fmol per tube. To account for the possibility of any nonspecific effects on the RIA, all drugs used in the release experiments were included in separate standard curves for the purposes of data analysis. We did not observe any alterations in the standard curve for any of the compounds utilized in these studies

### Data analysis and statistics

**Figure 11:**
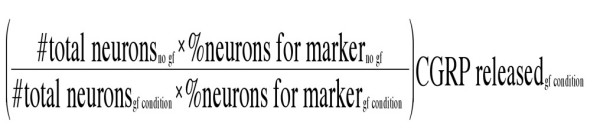
*Equation used to normalize CGRP release*. "No GF" refers to no growth factor controls and "GF condition" refers to any given growth factor treated condition

All data are presented as mean ± SEM unless otherwise stated. When normalizations for CGRP release or content to neuron numbers for either CGRP or TRPV1-immunoreactive neurons were made the equation shown in figure 11 was used. All data were analyzed using GraphPad Prism for Mac OSX (GraphPad, San Diego, CA). To assess statistical differences, data were analyzed by one-way ANOVA followed by Tukey's post-test, for multiple comparisons, or Dunnett's post-test to compare all groups to the control group. For differences between growth factors at the same concentrations differences were assessed by two-way ANOVA with Bonferroni post-test for between group comparisons. All nonlinear regressions were fit to a sigmoidal curve with variable slope.

## Abbreviations

ACEA: arachidonyl-2-chloroethylamide, AEA: anandamide, BDNF: brain-dervied neurotrphic factor, CAP: capsaicin, CGRP: calcitonin gene-related peptide, DRG: dorsal root ganglion, GDNF: glial cell-line derived neurotrphic factor, IB_4_: isolectin B_4_, ICC: immunocytochemistry, NGF: nerve growth factor, SP: substance P, TG: trigeminal ganglion, TRPV1, transient receptor potential receptor vanilloid type 1

## Author's Contributions

TJP performed and conceived (or participated in their conception) experiments in each section, analyzed all data and authored the manuscript. MDL performed immunocytochemistry and neuron counts. DCS conducted the intitial CAP-evoked CGRP release study. GOD assisted in conceiving the experiments and performing the pilot studies. NAJ conducted the Western blots. AP assisted in conducting the CGRP release experiments. AD assisted in real-time PCR experiments. AAT assisted in generating cultures and in conducting pilot studies for CAP-evoked CGRP release. KMH assisted in conceiving the experiments and gave critical readings of the manuscript. CMF supervised all studies and conceived the original experimental designs as well as critically editing the manuscript.

## Figures and Tables

**Table 1:** Percentage of neurons expressing markers.

	**X**	**N1**	**N10**	**N100**	**G1**	**G10**	**G100**	**B1**	**B10**	**B100**
**TRPV1**	37.9	37.5	37.4	40.0	48.4	57.5	66.6	50.1	50.9	47.8
**CGRP**	67.1	61.4	61.0	54.3	64.6	74.4	76.0	65.0	68.7	63.5
**IB_4_**	56.5	61.3	73.1	62.0	63.3	65.0	63.9	59.6	61.8	47.2

The percentage of total NF-H-positive neurons expressing TRPV1, CGRP or IB_4 _is shown for each growth factor condition (X: no growth factor, N: NGF, G: GDNF, B: BDNF).
